# Supplementary figures and images for: Hexokinase 2 is dispensable for photoreceptor development but is required for survival during aging and outer retinal stress
Source: Cell Death Dis. 2020 Jun 4;11(6):422. doi: 10.1038/s41419-020-2638-2 (PMC7272456; doi:10.1038/s41419-020-2638-2)

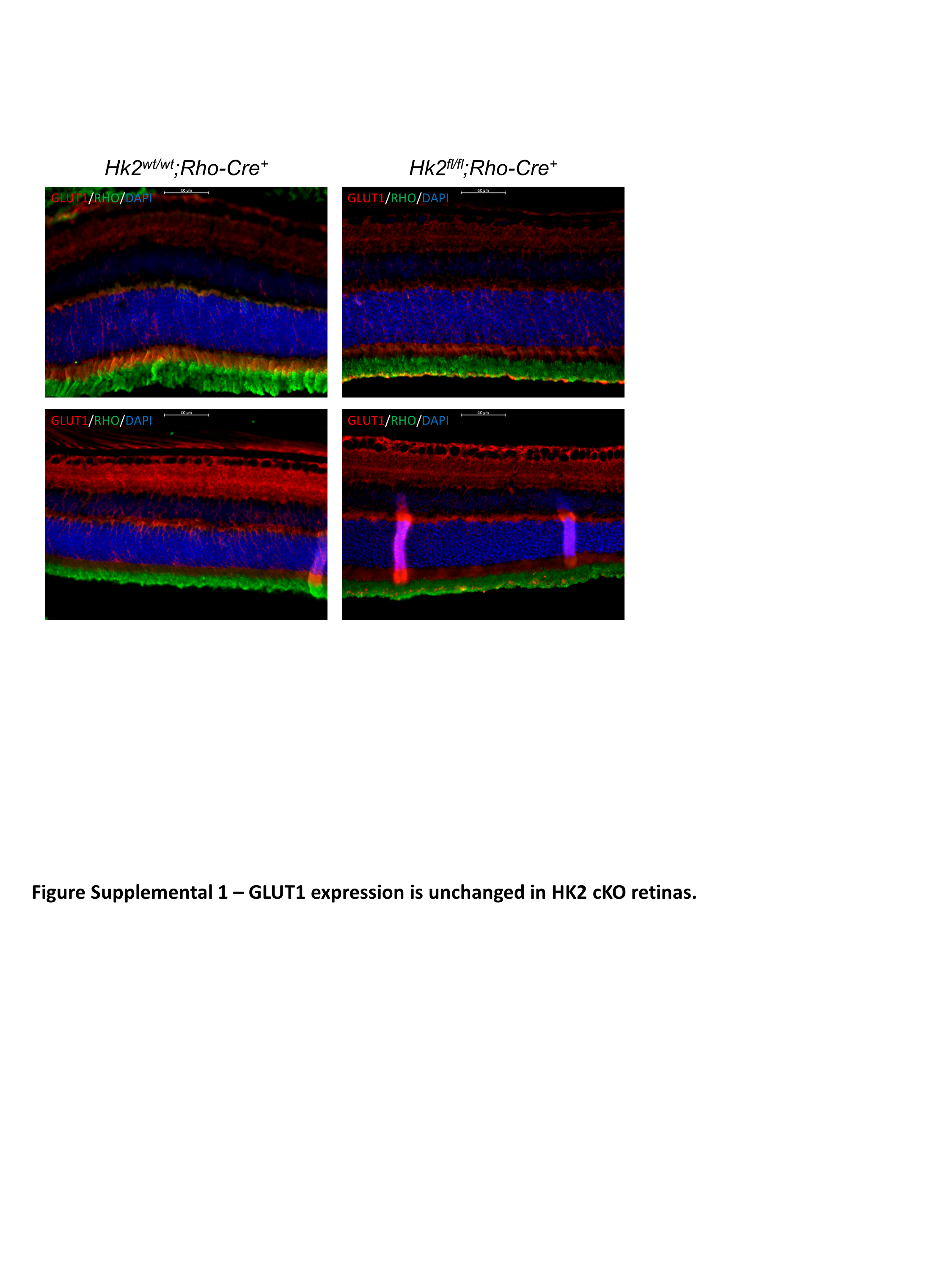

Supplement: Supplementary file 1 — Figure Supplemental 1 [file 41419_2020_2638_MOESM1_ESM.tif]
