# Supplementary material for: Hexokinase 2 is dispensable for photoreceptor development but is required for survival during aging and outer retinal stress
Source: Cell Death Dis. 2020 Jun 4;11(6):422. doi: 10.1038/s41419-020-2638-2 (PMC7272456; doi:10.1038/s41419-020-2638-2)
Supplement: Supplementary file 3 — Supplemental Figure Legends [file 41419_2020_2638_MOESM3_ESM.docx]

**Figure Supplemental 1 – GLUT1 expression is unchanged in HK2 cKO retinas.**

Representative immunofluorescent images showing that GLUT1 expression and localization is unchanged in the retina in cKO animals compared to WT animals. GLUT1 – glucose transporter 1, RHO – rhodopsin, DAPI - 4′,6-diamidino-2-phenylindole
